# Postabortion care availability, facility readiness and accessibility in Nigeria and Côte d’Ivoire

**DOI:** 10.1093/heapol/czab068

**Published:** 2021-05-29

**Authors:** Suzanne O Bell, Mridula Shankar, Saifuddin Ahmed, Funmilola OlaOlorun, Elizabeth Omoluabi, Georges Guiella, Caroline Moreau

**Affiliations:** Department of Population, Family and Reproductive Health, Johns Hopkins Bloomberg School of Public Health, 615 N. Wolfe Street, Baltimore, MD 62501, USA; Department of Population, Family and Reproductive Health, Johns Hopkins Bloomberg School of Public Health, 615 N. Wolfe Street, Baltimore, MD 62501, USA; Department of Population, Family and Reproductive Health, Johns Hopkins Bloomberg School of Public Health, 615 N. Wolfe Street, Baltimore, MD 62501, USA; College of Medicine, University of Ibadan, Queen Elizabeth II Road, Agodi, Ibadan, Nigeria; Center for Research, Evaluation Resources and Development, Flat 16, Ajanaku Estate Ife-Ibadon Road, Opp RCCG Rehoboth Mega Cathedral, Ile-Ife Osun State, Nigeria; Department of Statistics and Population Studies, University of the Western Cape, Robert Sobukwe Road, P/Bag X17, Bellville, 7530 Cape Town, South Africa; Institut Supérieur des Sciences de la Population (ISSP), Université of Ouagadougou, 03 BP 7118, Blvd Charles De Gaulle, Ouagadougou, Burkina Faso; Department of Population, Family and Reproductive Health, Johns Hopkins Bloomberg School of Public Health, 615 N. Wolfe Street, Baltimore, MD 62501, USA; Soins et Santé Primaire, CESP Centre for Research in Epidemiology and Population Health U1018, Inserm, Bat 15/16 16 av PV Couturier, 94807 Villejuif Cedex, France

**Keywords:** Postabortion care, abortion, Nigeria, Côte d’Ivoire, survey

## Abstract

Postabortion care (PAC) is an essential component of emergency obstetric care (EmOC) and is necessary to prevent unsafe abortion-related maternal mortality, but we know little regarding the preparedness of facilities to provide PAC services, the distribution of these services and disparities in their accessibility in low-resource settings. To address this knowledge gap, this study aims to describe PAC service availability, evaluate PAC readiness and measure inequities in access to PAC services in seven states of Nigeria and nationally in Côte d’Ivoire. We used survey data from reproductive-age women and the health facilities that serve the areas where they live. We linked facility readiness information, including PAC-specific signal functions, to female data using geospatial information. Findings revealed less than half of facilities provide basic PAC services in Nigeria (48.4%) but greater PAC availability in Côte d’Ivoire (70.5%). Only 33.5% and 36.9% of facilities with the capacity to provide basic PAC and only 23.9% and 37.5% of facilities with the capacity to provide comprehensive PAC had all the corresponding signal functions in Nigeria and Côte d’Ivoire, respectively. With regard to access, while ∼8 out of 10 women of reproductive age in Nigeria (81.3%) and Côte d’Ivoire (79.9%) lived within 10 km of a facility providing any PAC services, significantly lower levels of the population lived <10 km from a facility with all basic or comprehensive PAC signal functions, and we observed significant inequities in access for poor, rural and less educated women. Addressing facilities’ service readiness will improve the quality of PAC provided and ensure postabortion complications can be treated in a timely and effective manner, while expanding the availability of services to additional primary-level facilities would increase access—both of which could help to reduce avoidable abortion-related maternal morbidity and mortality and associated inequities.

**Key messages**

- Findings revealed less than half of facilities provide basic PAC services in Nigeria (48.4%) but greater PAC availability in Côte d’Ivoire (70.5%). However, not all facilities providing basic or comprehensive PAC had all the corresponding PAC signal functions, an indication of insufficient readiness to provide quality PAC.
- Results suggest that access to PAC is inadequate in both countries. While ∼8 out of 10 women of reproductive age in Nigeria (81.3%) and Côte d’Ivoire (79.9%) lived within 10 km of a facility providing any PAC services, significantly lower levels of the population lived <10 km from a facility with all basic or comprehensive PAC signal functions, and we observed significant inequities in access for poor, rural and less educated women.
- Efforts to expand availability and access to PAC are urgently needed to reduce avoidable abortion-related maternal morbidity and mortality and associated inequities.

## Background

Induced abortion is a common reproductive recourse for addressing unwanted pregnancies, regardless of legality. The global rate for the 2015-19 period was 39 abortions per 1000 women aged 15-49 years, with no significant difference in rates between countries by their legalization status (Bearak *et al.*, 2020). However, there are diverging trends in the rates between high- and low-resource countries. In high-resource settings, the abortion rate decreased significantly by 31% from 1990-94 to 2015-19, whereas the corresponding rates in low-resource settings were similar in both periods (Bearak *et al.*, 2020). Coupled with growing populations in the developing world, this corresponds to a growing share of global abortions occurring in low-resource settings; of the estimated 56.3 million annual abortions that occurred worldwide during the period 2010-14, 88% (49.6 million) occur in developing countries, an increase of >11 million abortions and 11 percentage points since the 1990–94 period (Sedgh *et al.*, 2016). These changing trends may slow the progress in global reductions in abortion-related mortality observed since the 1990s as a greater proportion of abortions are conducted in unsafe conditions in low-resource settings (Kassebaum *et al.*, 2014; Ganatra *et al.*, 2017).

While induced abortion is among the safest medical procedures when performed according to recommended guidelines (Grimes *et al.*, 2006), unsafe abortions undertaken by 25 million women annually currently cause between 8% and 15% of maternal deaths worldwide, nearly all of which occur in low-resource settings and countries where abortion is legally restricted (Kassebaum *et al.*, 2014; Say *et al.*, 2014; Ganatra *et al.*, 2017). The risks of mortality associated with these unsafe abortions vary widely across contexts, partly due to the extent of medication abortion drug diffusion outside of the healthcare system and also because of differential availability and quality of postabortion care (PAC) services. The case fatality associated with unsafe abortion in developing regions is 220 deaths per 100 000 unsafe abortions; this is in contrast to a case fatality rate of 30 per 100 000 in developed regions (World Health Organization, 2012). This is highly correlated with legality; even among developed countries, the case fatality rate for unsafe abortion is 40 times higher than that for legally induced abortions (World Health Organization, 2012). Thus, while abortion rates may not differ by legality (Guttmacher Institute, 2018), abortion-related mortality rates differ significantly. These disparities in abortion-related mortality are both a function of inequitable access to safe abortion procedures (primary prevention) and to quality PAC services to treat unsafe abortion complications (secondary prevention). The lack of primary prevention relates largely to the legal context, while lack of adequate secondary prevention refers to insufficient resources and poor quality of obstetric care in general and PAC services in particular. To reduce the negative outcomes associated with unsafe abortion, regardless of legality, PAC services—which are an essential component of emergency obstetric care (EmOC)—are necessary (Grimes *et al.*, 2006). As far back as 1994, the International Conference on Population and Development called for increased availability of PAC services, stating that ‘in all cases, women should have access to quality services for the management of complications arising from abortion’ (United Nations Population Fund, 2004).

Despite PAC’s importance in managing medical emergencies to prevent abortion-related maternal mortality, we know little regarding the preparedness of facilities to provide PAC services, the distribution of these services and the relationship between PAC availability and women’s abortion care-seeking and safety in many low-resource settings. Recent evidence from several low-resource countries suggests that PAC availability is mostly scarce (Owolabi *et al.*, 2019). Conclusions from a systematic review of PAC service provision in sub-Saharan Africa corroborate these findings, indicating that while PAC services have been expanding in recent years, quality remains an issue and myriad barriers continue to limit access to and use of these services (Izugbara *et al.*, 2019). The extent to which facilities are able to and actually provide PAC is important to understand and can inform the need for additional scale-up efforts; however, these data are lacking for many countries (Izugbara *et al.*, 2019).

Healthcare service ‘readiness’, or a facility’s capacity to provide all components of a service, is an essential aspect of quality of care. Service readiness is a necessary precursor to achieving quality of care, which is a broader although fundamental function of a healthcare system (Kruk *et al.*, 2018). Investigators have long viewed facility-based healthcare services in terms of Donabedian’s three dimensions of quality: structural, process and outcome (Donabedian, 1966). Key elements required to provide a health service, which typically include both structural and process elements of quality, are called signal functions. Signal functions for safe abortion care (SAC) are organized in a framework that includes three components: safe-induced abortion for all legal indications, treatment of abortion complications and provision of postabortion contraception (Healy *et al.*, 2006). The framework distinguishes between basic and comprehensive SAC: basic SAC involves the capacity to provide abortion and PAC at early gestations (12 weeks or less) and a related constellation of services for uncomplicated cases, whereas comprehensive SAC requires a facility to also be able to provide these services at later gestations (beyond 12 weeks), treat serious complications and provide counselling and services for long-acting or permanent postabortion contraceptive methods.

Existing research has used the signal functions to assess service-specific preparedness of health facilities, but these studies lack a holistic, nuanced measure of SAC or PAC readiness. For instance, the limited prior research on SAC and PAC readiness has focused on the availability of ‘individual’ signal functions or a composite measure of the percentage of facilities with ‘all’ basic or comprehensive signal functions; this approach generally does not distinguish between facilities with no signal functions and those with all but one (Bell *et al.*, 2017; Owolabi *et al.*, 2019; Campbell *et al.*, 2016; Otsea *et al.*, 2011; Abdella *et al.*, 2013; Huda *et al.*, 2015). This is a hindrance to a more in-depth assessment of the extent of efforts required for facilities to meet all signal functions. However, existing findings using available signal function measures indicate that facility capacity to provide PAC in most low-resource countries is inadequate (Owolabi *et al.*, 2019).

The current study focuses on PAC in Nigeria and Côte d’Ivoire. These countries were selected as part of a three-country parent study (also conducted in Rajasthan, India), which sought to estimate abortion incidence and safety in these context in order to address the data gap in abortion indicators, particularly in West Africa (Ahmad *et al.*, 2020; Bell *et al.*, 2019; 2020a,b,c). In most states in Nigeria and nationally in Côte d’Ivoire, abortion is only legal to save a woman’s life, yet unsafe illegal abortion—and its consequences—is common. Maternal mortality in these countries is high, with estimates ranging from 500 to nearly 1000 deaths per 100 000 live births (Hogan *et al.*, 2010; Kassebaum *et al.*, 2014; World Health Organization, 2015; National Population Commission (NPC) [Nigeria] & ICF 2019). Research suggests that 10–18% of these deaths are a result of unsafe abortion, which have a case fatality rate in West Africa of 540 deaths per 100 000 unsafe abortions; this is 800 times higher than the case fatality rate associated with legal abortion in the USA (Kassebaum *et al.*, 2014; Say *et al.*, 2014; World Health Organization, 2012). For each death, there are hundreds of women who experience severe and potentially life-threatening complications (Akinlusi *et al.*, 2018).

Despite these risks, abortion remains a common means of preventing unwanted births in these countries. Findings from a recent national study in Côte d’Ivoire suggest a 1-year incidence of 41 abortions per 1000 women of reproductive age (Bell *et al.*, 2020c). Abortion is similarly common in Nigeria, with the 1-year incidence recently estimated at 46 per 1000 women of reproductive age (Bell *et al.*, 2020a). Nearly two-thirds of these abortions in both countries are highly unsafe, involving non-recommended methods and non-clinical sources (Bell *et al.*, 2019). Among gynaecological admissions at a Nigerian teaching hospital in recent years, investigators found that 7.4% were related to the treatment of unsafe abortion, 17% of which ultimately resulted in maternal death (Akinlusi *et al.*, 2018). While recent studies in these countries suggest the common occurrence of unsafe abortion, which in some cases may require additional care for complications, no prior published studies have documented the availability and readiness of PAC services for secondary prevention.

To address this knowledge gap, the first aim of this study is to describe PAC service availability and evaluate PAC readiness among seven states in Nigeria and nationally in Côte d’Ivoire. Our analysis focuses primarily on PAC services given the legal context of the study settings where safe abortions are rarely legally permitted. The second aim is to determine the proportion of PAC patients receiving care from facilities with all basic and comprehensive PAC signal functions. The third aim is to measure inequities in access to PAC services by linking facility data with population-based data on reproductive-age women served by sampled facilities. Together, our results will provide a comprehensive appraisal of PAC service availability, readiness, and accessibility in these countries and clear points of intervention to improve health equity in relation to this life-saving service.

## Methods

Data for this study come from Performance Monitoring and Accountability 2020 (PMA2020), Nigeria and Côte d’Ivoire. (PMA2020) uses mobile-assisted technology to implement low-cost, rapid turnaround national/regional family planning monitoring surveys annually (Performance Monitoring for Action (PMA) 2021; Zimmerman *et al.*, 2017). In each country, a cadre of sentinel resident interviewers collect data at the household, individual and facility levels. The data we used for this study are cross-sectional and include surveys of service delivery points (SDPs) that serve a nationally representative population of reproductive-age women (15–49 years). In-country ethical review boards and the Johns Hopkins Bloomberg School of Public Health provided ethical approval for this study.

The sampling for the female survey in both countries employed a multi-stage cluster design with probability proportional to size (PPS) sampling of enumeration areas (EAs) within urban/rural geography strata. In Nigeria, PMA2020 was originally implemented in two states, Lagos and Kaduna. As demand for nationally representative female data increased, PMA2020 investigators selected an additional five states using PPS. Overall, two states were selected from the North West where 25% of the country’s population lives and one state from each of five remaining six geopolitical zones. EAs were selected from urban/rural-state strata. In both Nigeria and Cote d’Ivoire, EAs are geographic units comprised of ∼200 households and are defined by the central statistical or census office of the country. A sample of 35 households from each EA (40 for EAs in Lagos state, Nigeria) was randomly selected, and all women of reproductive age (15–49 years) from the selected households were invited to participate. Women provided verbal informed consent prior to participating in both countries. In Nigeria and Côte d’Ivoire, the household response rates were 97.5% and 97.6%, respectively, and the female response rates were both 98.1%.

At the same time, a sample of SDPs was created that included private and public SDPs serving the selected EAs. The sample was selected from a list of public sector facilities serving the geography, which we obtained from the local health authorities, and a list of all private SDPs within each EA, which interviewers created through mapping and listing. Up to three private SDPs were randomly selected per EA as well as up to three public facilities assigned to serve those EAs and representing each level of care (primary, secondary and tertiary). On average, each EA is served by less than one private SDP, and two to three public SDPs are designated as primary, secondary or tertiary levels of care for that area. The final SDP sample (excluding pharmacies and chemists) involved facilities that could potentially provide at least basic PAC. The SDP response rates in Nigeria and Côte d’Ivoire were 96.6% and 97.0%, respectively.

We linked the SDP sample to the female data using geospatial data. For each woman, we used the central point of her EA as her Global Positioning System (GPS) point as we did not have ethical review board approval to use individual women’s GPS data in Nigeria. As such, when we present findings on access, we are presenting the percent of women who live in an EA in which the midpoint is within 10 km of a given facility. For simplicity, in the ‘Results’ section of this paper, we refer to these findings as ‘the percent of women living within 10 km of a given facility’. For each SDP, we used the GPS point taken at the time of the interview. Neither the EA GPS points nor the facility GPS points were displaced. We then linked each woman to each SDP sampled using Euclidean distance and determined the distance to the closest sampled facility that provided any PAC, provided PAC and had all basic PAC signal functions, and provided PAC and had all comprehensive PAC signal functions. In total, 11 106 and 2738 women completed the survey in Nigeria and Côte d’Ivoire, respectively. One urban EA in Nigeria had inaccurate GPS data; thus, we excluded the 24 women from this EA, resulting in an analytic sample of 11 082 women. The 24 excluded women were slightly older, more educated and more likely to have average wealth than women nationally. All women in Côte d’Ivoire had GPS data.

To distinguish facility type, we divided facilities by level (referral vs primary) and sector (public vs private). In Nigeria, these categories corresponded to public referral (teaching hospitals, state hospitals and higher-level maternity centres), public primary (all lower-level facilities), private referral (tertiary hospitals and secondary hospitals) and private primary. Nigeria has a three-tier health system in the public sector; thus, the public referral category encompasses tertiary (or specialist) facilities managed by the federal government and secondary facilities managed by state governments; all public primary facilities are managed by local governments. In Côte d’Ivoire, we grouped facilities into public referral (teaching, regional and general hospitals), public primary (all lower-level facilities) and private primary (there were no referral-level private facilities in our Côte d’Ivoire sample). These categories were determined in conjunction with in-country partners based on the local healthcare system. Public and private referral facilities in Nigeria and public referral facilities in Côte d’Ivoire should have the capacity to provide comprehensive PAC; all primary facilities should have the capacity to provide basic PAC. Some primary facilities may potentially have the capacity to provide comprehensive PAC if a trained provider is present, but since this is rare, we assumed primary-level facilities would not be expected to provide this service. The one exception to excluding primary facilities from analyses of comprehensive PAC was in the context of assessing individual signal functions. We present the estimates of individual comprehensive PAC signal functions at primary facilities to show the extent to which these services are available even at some lower-level facilities, offering a more complete assessment of PAC readiness.

The SDP survey covered structural features of the facility, provider information, family planning service availability, stockouts and patient caseloads. Specific to this study, we included an additional module on abortion and postabortion services. We assessed facility abortion service readiness by measuring signal functions necessary to provide basic and comprehensive PAC services (Table 1). We created an index that combined PAC signal function information for each level of care (basic and comprehensive). The index is additive, providing a more nuanced measure of basic and comprehensive PAC readiness than a simple ‘all or nothing’ measure. We refer to this index as a readiness score, which we converted into a percentage that ranged from 0 to 100, representing the percent of signal functions a given facility has. PAC caseloads were reported by the person at the facility most knowledgeable about PAC and abortion service delivery. These respondents provided separate estimates of the number of inpatient and outpatient PAC clients treated in the last completed month and the average month. We averaged these two numbers to account for potential seasonality and multiplied by 12 to get annual PAC caseload estimates for each facility.

**Table 1.** Basic and comprehensive PAC signal functions criteria

Basic
≤12 weeks’ removal of retained products
Antibiotics
Oxytocics
Intravenous replacement fluids
Any contraception
Comprehensive (basic +)
>12 weeks’ removal of retained products
Blood transfusion
Laparotomy
24/7 PAC services available
Long-acting reversible contraception

Using the public SDP data and a sampling frame of public facilities provided by the government, we constructed public facility weights for each country that are the inverse of the probability of selection of each facility type (within each state in Nigeria) multiplied by the response rate for that facility type/state stratum. In Côte d’Ivoire, we had a sampling frame for private facilities as well, which we used to construct weights in a similar fashion. Since there was not a private facility sampling frame for Nigeria, we multiplied the household sample EA probabilities of selection and the response rate of that facility type within that EA and took the inverse to construct the private facility weights. With the weighted data, we sought to produce service readiness estimates that reflect the facility type distribution (among seven states in Nigeria and nationally in Côte d’Ivoire).

We conducted a number of analyses to assess PAC availability, readiness and accessibility in Nigeria and Côte d’Ivoire. To determine PAC availability, we used SDP respondent reports of whether the facility provided the service. We evaluated PAC readiness in a number of ways, including the percentage of all facilities with each basic and comprehensive signal function by facility type; the percentage of facilities with ‘all’ basic and comprehensive signal functions by facility characteristics (i.e. type, sector, as well as state in Nigeria) and the basic and comprehensive readiness score among PAC providing facilities by facility characteristics. We then estimated the percentage of PAC patients receiving care in facilities meeting basic and comprehensive readiness criteria, overall and by facility characteristics. Lastly, we determined accessibility by estimating the proportion of reproductive-age women living within a 10-km radius of a facility providing any PAC services, a facility with all basic PAC readiness criteria, as well as a facility with all comprehensive PAC readiness criteria, and explored potential sociodemographic characteristics associated with a lack of PAC accessibility within 10 km through bivariate and multivariate logistic regression. We applied survey weights that account for the complex sampling design and clustering—including the probability of selection and response rate—to the female data for the final analysis. We conducted all analyses in Stata version 15.1 (Statacorp, 2017).

## Results

### Facility characteristics

In total, we completed surveys with 429 facilities that had the potential to provide at least basic PAC in Nigeria and 129 in Côte d’Ivoire, the majority of which were public, primary facilities (Table 2). Among facilities in Nigeria, 6.4% were public referral, 84.3% were public primary, while 5.0% were private referral and 4.3% were private primary. The corresponding distribution in Côte d’Ivoire was 4.5% public referral, 79.9% public primary and 15.6% private primary. In Nigeria, 53.9% of private facilities were secondary and 46.1% were primary; we only captured primary private facilities in the Côte d’Ivoire sample. Among the 115 facilities that could provide comprehensive PAC in Nigeria (i.e. secondary- or tertiary-level facilities), 56.1% were public referral facilities; all of the 48 facilities with the capacity to provide comprehensive PAC in Côte d’Ivoire were public referral.

**Table 2.** Facility characteristics by whether facility eligible to provide basic or comprehensive PAC, Nigeria and Côte d’Ivoire^a^

	Nigeria				Côte d’Ivoire			
	Basic PAC		Comprehensive PAC		Basic PAC		Comprehensive PAC	
	**%**	***n***	**%**	***n***	**%**	***n***	**%**	***n***
Facility type								
Public referral	6.4	101	56.1	101	4.5	48	100.0	48
Public primary	84.3	285	–	–	79.9	67	–	–
Private referral	5.0	14	43.9	14	–	–	–	–
Private primary	4.3	29	–	–	15.6	14	–	–
Managing authority								
Public	90.7	386	56.1	101	84.4	115	100.0	48
Private	9.3	43	43.9	14	15.6	14	–	–
State (Nigeria)								
Anambra	9.7	62	12.5	6	–	–	–	–
Kaduna	25.2	88	29.2	23	–	–	–	–
Kano	20.2	46	11.0	17	–	–	–	–
Lagos	11.0	86	26.1	23	–	–	–	–
Nasarawa	14.3	61	4.9	9	–	–	–	–
Rivers	9.5	51	12.4	20	–	–	–	–
Taraba	10.1	35	3.9	7	–	–	–	–
Total	100.0	429	100.0	115	100.0	129	100.0	48

a Percentage values weighted, *n* values unweighted.

### PAC service availability

Findings revealed low levels of PAC service provision at earlier gestational ages (≤12 weeks)—a key signal function for basic PAC—among all facilities with the capacity to provide basic PAC in Nigeria (48.4%), this proportion rising to 70.5% in Côte d’Ivoire. In facilities with the capacity to provide comprehensive PAC, 82.2% and 87.5% provided PAC for later (>12 weeks) gestations—a key signal function for comprehensive PAC—in Nigeria and Côte d’Ivoire, respectively (Table 3). Overall PAC availability was driven by the high availability of services at earlier gestations (i.e. basic PAC) in public referral facilities, where 80.4% of these facilities in Nigeria and 93.8% in Côte d’Ivoire offered PAC. There were much more limited early PAC services at public and private primary-level facilities in Nigeria (43.3% and 49.9%, respectively) and Côte d’Ivoire (74.6% and 42.9%, respectively). However, because primary care services are more numerous, public primary facilities represent 75.3% of facilities with PAC services in Nigeria and 84.6% in Côte d’Ivoire (estimates not shown in the table).

**Table 3.** Percentage of all facilities offering PAC at ≤12-week and >12-week gestation and average PAC caseload by facility characteristics, Nigeria and Côte d’Ivoire^a^

	Nigeria				Côte d’Ivoire			
	≤12 weeks	>12 weeks	Provided PAC last month	Avg. monthly caseload^b^	≤12 weeks	>12 weeks	Provided PAC last month	Avg. monthly caseload^b^
*n*	429	115	429	214	129	48	129	101
Facility type								
Public referral	80.4	73.9	65.1	16.0 (2.4)	93.8	87.5	81.3	15.1 (1.9)
Public primary	43.3	–	27.2	5.0 (0.8)	74.6	–	44.8	3.8 (1.6)
Private referral	92.9	92.9	81.4	4.7 (1.7)	–	–	–	–
Private primary	49.9	–	35.1	4.0 (1.5)	42.9	–	21.4	5.2 (1.6)
Managing authority								
Public	45.9	73.9	29.9	7.0 (0.8)	75.6	87.5	46.7	5.4 (1.4)
Private	73.1	92.9	60.1	4.5 (1.3)	42.9	–	21.4	5.2 (1.6)
State (Nigeria)								
Anambra	32.2	57.7	11.6	3.1 (1.1)	–	–	–	–
Kaduna	54.5	88.1	34.2	4.1 (0.5)	–	–	–	–
Kano	40.1	73.8	33.4	11.0 (2.6)	–	–	–	–
Lagos	50.4	98.5	36.1	7.0 (1.7)	–	–	–	–
Nasarawa	42.0	88.9	23.7	7.5 (3.0)	–	–	–	–
Rivers	47.2	62.9	23.0	3.7 (1.1)	–	–	–	–
Taraba	73.4	85.7	66.1	7.9 (2.0)	–	–	–	–
Total	48.4	82.2	32.7	6.5 (0.7)	70.5	87.5	42.8	5.3 (1.1)

a Results weighted to account for probability of selection and response rate (within each state in Nigeria).

b Among facilities that report providing any PAC/SAC; mean (SE).

By sector, we observed much lower levels of PAC service availability among public facilities in Nigeria but significantly higher availability among public facilities in Côte d’Ivoire. In both sites, the majority of facilities that offered PAC had provided it in the month prior (67.6% in Nigeria, 60.6% in Côte d’Ivoire) (estimates not shown in the table). With regard to caseload, facilities treated on average between 4 and 16 PAC patients per month, with referral level public facilities in both contexts treating three to four times as many PAC patients as lower-level facilities; the difference in PAC caseloads by facility level was much smaller among private facilities in Nigeria and there were no private referral facilities in Côte d’Ivoire (Table 3). Public facilities also treated more PAC patients per month on average than private facilities in Nigeria; however, average facility caseloads were similar by sector in Côte d’Ivoire. In both settings, there were far fewer private facilities relative to the number of public facilities; thus, the public sector treated a much larger share of PAC cases overall despite relatively similar average monthly caseloads.

### PAC signal functions and service readiness

Among all facilities with the capacity to provide basic PAC, there was wide variability in the availability of PAC signal functions across facility types (Table 4). While 48.4% of all facilities in Nigeria and 70.5% of all facilities in Côte d’Ivoire reported offering PAC for early gestation pregnancies (≤12 weeks’ removal of retained produces of conception), only 43.9% of facilities in Nigeria and 69.2% of facilities in Côte d’Ivoire reported having at least one provider formally trained in PAC provision, with the largest deficits at the primary levels. Availability of oxytocics and intravenous replacement fluids was also among the most common missing basic signal functions (Table 4). In both countries, private primary facilities also had a low availability of any contraception. Laparotomy was the most common missing comprehensive signal function in both countries, followed by blood transfusion and 24/7 PAC service availability (Table 4).

**Table 4.** Percentage of all facilities that have specific components for basic and comprehensive PAC, Nigeria and Côte d’Ivoire^a^

	Nigeria					Côte d’Ivoire				
	Public referral	Public primary	Private referral	Private primary	Total	Public referral	Public primary	Private referral	Private primary	Total
*n*	101	285	14	29	429	48	67	0	14	129
Basic										
≤12 weeks’ removal of retained products of conception	80.4	43.3	92.9	49.9	48.4	93.8	74.6	–	42.9	70.5
At least one provider formally trained in PAC^b^	75.1	38.6	92.9	43.9	43.9	89.6	74.6	–	35.7	69.2
Antibiotics	97.5	96.2	98.7	97.5	96.4	97.9	100.0	–	92.9	98.8
Oxytocics	84.2	58.1	85.3	70.6	61.7	93.8	73.1	–	42.9	69.3
Intravenous replacement fluids	89.2	66.3	86.3	84.2	69.6	85.4	58.2	–	50.0	58.1
Any contraception	91.9	91.1	86.6	61.2	89.6	97.9	98.5	–	42.9	89.8
Comprehensive (basic +)										
>12 weeks’ removal of retained products of conception	73.9	26.8	92.9	43.4	33.8	87.5	50.7	–	28.6	48.9
Blood transfusion	82.3	19.5	75.3	43.8	27.4	85.4	6.0	–	0.0	8.6
Laparotomy	60.1	4.8	42.2	34.2	11.5	54.2	0.0	–	7.1	3.5
24/7 PAC services available	42.3	17.6	72.7	36.8	22.8	83.3	44.8	–	21.4	42.9
Long-acting reversible contraception	88.2	66.3	73.5	44.5	67.1	95.8	86.6	–	21.4	76.8

a Public facility results weighted; private facility results unweighted. Results weighted to account for probability of selection and response rate (within each state in Nigeria).

b Excluded from estimates of index of basic/comprehensive signal functions.

Percent of facilities with all PAC signal functions and percent of each signal function present varied by facility characteristics (Table 5). Only 33.5% of Nigerian and 36.9% of Ivoirian facilities with the capacity to provide basic PAC had all basic PAC signal functions. Among facilities with the capacity to provide comprehensive PAC, only 23.9% and 37.5% had all the corresponding signal functions in Nigeria and Côte d’Ivoire, respectively. The more nuanced signal function readiness score revealed that many facilities that provide PAC but do not have all signal functions are close to having all of them. Facilities that reported providing PAC in Nigeria and Côte d’Ivoire had on average ∼90% of basic signal functions; facilities with the capacity to provide comprehensive PAC that were providing PAC similarly had close to 90% of signal functions in both settings (Table 5). We observed variability by facility type in both countries, with higher percentages of signal functions present at higher-level facilities; this was particularly true when examining the percent with ‘all’ signal functions but less pronounced when examining the signal functions readiness score. Signal function availability was consistently higher in the public than in the private sector in Côte d’Ivoire regardless of whether examining the availability of all signal functions or the readiness score (Table 5). This was also true for comprehensive PAC signal functions for higher-level facilities in Nigeria. In contrast, all basic PAC signal functions were more likely to be available in private referral facilities than in public referral facilities in Nigeria while the primary-level facilities had similar levels by managing authority (Table 5). Readiness scores in Nigeria were similar across sectors by facility type (Table 5).

**Table 5.** Percentage of facilities that have all basic and comprehensive PAC signal functions by facility characteristics, Nigeria and Côte d’Ivoire^a^

	Nigeria				Côte d’Ivoire			
	Basic PAC		Comprehensive PAC		Basic PAC		Comprehensive PAC	
	Service availability	Readiness score	Service availability	Readiness score	Service availability	Readiness score	Service availability	Readiness score
	All facilities: % with all basic	PAC providing facilities: basic score^b^	All facilities: % with all comp	PAC providing facilities: comp score^b^	All facilities: % with all basic	PAC providing facilities: basic score^b^	All facilities: % with all comp	PAC providing facilities: comp score^b^
*n*	429	214	115	92	129	101	48	45
Facility type								
Public referral	67.6	96	30.8	89	79.2	96	37.5	90
Public primary	28.4	91	–	–	40.3	89	–	–
Private referral	80.5	93	15.0	84	–	–	–	–
Private primary	27.8	90	–	–	7.1	83	–	–
Managing authority								
Public	31.2	91	30.8	89	42.4	90	37.5	90
Private	56.2	92	15.0	84	7.1	83	–	–
State								
Anambra	20.5	87	24.1	87	–	–	–	–
Kaduna	40.7	93	8.4	90	–	–	–	–
Kano	20.1	86	31.8	89	–	–	–	–
Lagos	38.3	91	31.0	83	–	–	–	–
Nasarawa	30.8	95	55.6	95	–	–	–	–
Rivers	33.9	93	19.3	81	–	–	–	–
Taraba	52.9	92	42.9	90	–	–	–	–
Total	33.5	91	23.9	87	36.9	89	37.5	90

a Results weighted to account for probability of selection and response rate (within each state in Nigeria).

b Readiness score from 0 to 100.

Based on reported caseload data, we found that 92.1% of PAC patients in Nigeria and 89.7% of PAC patients in Côte d’Ivoire were treated in facilities with all basic PAC signal functions (Table 6). Around half of PAC patients in Nigeria (54.5%) and Côte d’Ivoire (47.3%) received care at a facility with all comprehensive signal functions. Corresponding to the aforementioned PAC service availability and readiness findings, PAC patients treated in higher-level facilities were more likely to receive care at a facility with all basic or comprehensive signal functions. By sector, we saw slightly lower percentages of PAC patients treated in private facilities with all basic signal functions in Nigeria but much larger differences in Côte d’Ivoire; the pattern was reversed with regard to comprehensive PAC in Nigeria with a higher percentage of PAC caseloads treated in private versus public facilities having all comprehensive signal functions (Table 6). Additionally, there was substantial variability by state in Nigeria, with 100.0% of PAC patients in Lagos treated in facilities with all basic signal functions compared to 63.5% of PAC patients in Anambra; the percentage of PAC patients treated in facilities with all comprehensive signal functions ranged from 26.0% in Kaduna to 89.3% in Nasarawa (Table 6).

**Table 6.** Percent of PAC caseloads treated by facilities with 100% of basic or comprehensive signal functions among facilities that offer PAC, by facility characteristics, Nigeria and Côte d’Ivoire^a^

	Nigeria		Côte d’Ivoire	
	Basic	Comp	Basic	Comp
*n*	429	115	129	48
Facility type				
Public referral	96.6	55.9	93.2	47.3
Public primary	74.2	–	80.7	–
Private referral	96.5	65.5	–	–
Private primary	78.0	–	46.2	–
Managing authority				
Public	92.4	54.0	91.8	47.3
Private	88.2	65.5	46.2	–
State				
Anambra	63.5	52.9	–	–
Kaduna	93.2	26.0	–	–
Kano	88.4	32.8	–	–
Lagos	100.0	66.0	–	–
Nasarawa	98.9	89.3	–	–
Rivers	86.4	55.5	–	–
Taraba	77.8	44.5	–	–
Total	92.1	54.5	89.7	47.3

a Results weighted to account for the probability of selection and response rate (within each state in Nigeria).

### Access to PAC services among women of reproductive age

Approximately 8 out of 10 women of reproductive age in Nigeria (81.3%) and Côte d’Ivoire (79.9%) lived within 10 km of a facility providing any PAC services, while 71.6% and 63.5% lived <10 km from a facility with all basic PAC signal functions, and only 42.4% and 25.1% lived within 10 km of a facility with all comprehensive PAC signal functions (Table 7). Women who had never attended school had the lowest level of geographic access to facilities with basic PAC readiness in Nigeria (42.4%) and Côte d’Ivoire (53.5%) compared to those with higher education (91.2% and 93.8%, respectively) (Table 7). Urban residents in Nigeria and Côte d’Ivoire were significantly more likely to live within 10 km of a facility with all basic signal functions (94.0% and 82.0%, respectively) compared to rural residents (41.8% and 33.9%). The poorest women in Nigeria (40.6%) and Côte d’Ivoire (30.8%) were also significantly less likely to live near a facility with basic PAC readiness compared to the wealthiest women (95.0% and 93.6%) (Table 7). These patterns were similar when assessing access to facilities with all comprehensive signal functions; however, the percentage of women living near facilities that met the comprehensive criteria, regardless of characteristics, was lower (Table 7). In Nigeria, there were also substantial differences in access to PAC services by state, regardless of criteria examined.

**Table 7.** Percent of women in Nigeria and Côte d’Ivoire living within 10 km of a facility offering any PAC or with all basic or comprehensive PAC signal functions, by background characteristics^a^

	Nigeria				Côte d’Ivoire			
	*n*	Any	Basic	Comprehensive	*n*	Any	Basic	Comprehensive
Age (years)								
15–19	2255	80.9	70.8	42.3	542	83.3	67.9	28.6
20–24	1869	80.9	70.0	41.9	500	81.7	63.2	19.9
25–29	2036	80.5	71.2	41.7	495	81.4	64.3	24.3
30–34	1625	81.8	73.5	43.6	436	77.8	61.8	26.5
35–39	1466	82.0	73.3	41.9	351	78.4	63.2	27.7
40–44	1097	81.2	71.1	43.1	262	75.9	61.0	25.2
45–49	734	82.8	71.2	43.6	152	72.4	55.0	21.9
Education								
None	2353	**64.6**	**42.4**	**24.8**	1254	**72.0**	**53.5**	**17.3**
Primary	1902	**71.2**	**56.9**	**33.8**	714	**79.7**	**60.6**	**22.8**
Secondary	4917	**85.2**	**78.7**	**46.0**	615	**90.4**	**78.4**	**35.4**
Higher	1910	**94.2**	**91.2**	**55.9**	152	**100.0**	**93.8**	**52.6**
Residence								
Rural	5701	**63.4**	**41.8**	**13.3**	1062	**56.9**	**33.9**	**8.0**
Urban	5381	**94.8**	**94.0**	**64.4**	1676	**94.2**	**82.0**	**35.8**
Wealth tertile								
Poorest	4931	**62.3**	**40.6**	**17.7**	854	**51.1**	**30.8**	**6.3**
Middle	3273	**88.6**	**83.6**	**56.4**	934	**87.4**	**62.8**	**19.3**
Wealthiest	2878	**95.8**	**95.0**	**57.3**	950	**99.4**	**93.6**	**47.0**
State								
Anambra	1419	**96.3**	**96.3**	**32.7**	–	–	–	–
Kaduna	2766	**72.1**	**61.3**	**33.2**	–	–	–	–
Kano	1419	**69.2**	**54.2**	**49.0**	–	–	–	–
Lagos	2766	**92.4**	**92.4**	**92.4**	–	–	–	–
Nasarawa	1751	**59.2**	**49.3**	**25.3**	–	–	–	–
Rivers	1566	**95.1**	**81.6**	**14.6**	–	–	–	–
Taraba	1536	**71.9**	**47.7**	**25.0**	–	–	–	–
Total	11 082	81.3	71.6	42.4	2738	79.9	63.5	25.1

a Bold values indicate statistically significant difference at the *P* < 0.05 level.

Multivariate logistic regression results showed that higher wealth was independently associated with increased odds of residing <10 km from a facility with any PAC and those with all basic or comprehensive PAC signal functions in Nigeria (Table 8) and Côte d’Ivoire (Table 9). In Nigeria specifically, although some confidence intervals (CIs) were very wide, women living in urban areas had more than seven times the odds of living near a facility that met each of the PAC criteria, while the wealthiest women had two to more than five times the odds of living near a facility that met any of the PAC criteria compared to women in the lowest wealth category. State was also related to proximity to facilities with all basic and comprehensive PAC signal functions in Nigeria. In Côte d’Ivoire, while some CIs were again very wide, odds of proximity to PAC services for wealthier women were upwards of 2 to more than 46 times that of women in the lowest wealth category (Table 9).

**Table 8.** Adjusted odds ratio of living within 10 km of a facility providing any PAC, PAC with all basic signal functions and PAC with all comprehensive signal functions among reproductive-age women in Nigeria (*n* = 11 082)

	Any PAC		Basic PAC		Comprehensive PAC	
	aOR^a^	95% CI	aOR^a^	95% CI	aOR^a^	95% CI
Age (years)						
15–19	1.00	1.00, 1.00	1.00	1.00, 1.00	1.00	1.00, 1.00
20–24	1.03	0.81, 1.32	1.07	0.85, 1.33	1.15	0.92, 1.44
25–29	0.82	0.58, 1.17	0.87	0.63, 1.20	0.94	0.73, 1.22
30–34	0.77*	0.57, 1.03	0.9	0.67, 1.21	0.91	0.69, 1.20
35–39	0.8	0.58, 1.11	0.93	0.68, 1.27	0.77*	0.58, 1.02
40–44	0.92	0.66, 1.29	1.04	0.76, 1.42	0.96	0.73, 1.25
45–49	0.88	0.61, 1.26	0.88	0.64, 1.21	1.05	0.77, 1.45
Education						
None	1.00	1.00, 1.00	1.00	1.00, 1.00	1.00	1.00, 1.00
Primary	0.76	0.48, 1.21	0.92	0.59, 1.45	0.9	0.55, 1.46
Secondary	0.77	0.42, 1.39	1.06	0.59, 1.89	0.99	0.53, 1.86
Higher	1.15	0.62, 2.13	1.22	0.68, 2.20	1.1	0.59, 2.07
Residence						
Rural	1.00	1.00, 1.00	1.00	1.00, 1.00	1.00	1.00, 1.00
Urban	7.51***	2.21, 25.48	13.65***	4.88, 38.22	8.34***	3.03, 22.95
Wealth tertile						
Poorest	1.00	1.00, 1.00	1.00	1.00, 1.00	1.00	1.00, 1.00
Middle	1.7	0.86, 3.35	2.18**	1.20, 3.95	3.52***	2.05, 6.01
Wealthiest	2.95*	0.98, 8.88	5.26***	1.90, 14.56	2.47**	1.13, 5.36
State						
Anambra	1.00	1.00, 1.00	1.00	1.00, 1.00	1.00	1.00, 1.00
Kaduna	0.21**	0.05, 0.88	0.14***	0.03, 0.62	2.6	0.57, 11.87
Kano	0.18**	0.04, 0.83	0.10***	0.02, 0.44	7.82***	2.28, 26.88
Lagos	0.14**	0.02, 0.95	0.08***	0.01, 0.54	17.11***	3.66, 79.99
Nasarawa	0.18**	0.03, 0.90	0.15**	0.03, 0.78	3.51	0.69, 17.74
Rivers	1.01	0.15, 6.95	0.17**	0.04, 0.80	0.36	0.09, 1.48
Taraba	0.38	0.07, 2.07	0.19**	0.04, 0.97	5.17**	1.15, 23.29

*P < 0.10, **P < 0.05, ***P < 0.01.

a Adjusted odds ratio.

**Table 9.** Adjusted odds ratio of living within 10 km of a facility providing any PAC, PAC with all basic signal functions and PAC with all comprehensive signal functions among reproductive-age women in Côte d’Ivoire (*n* = 2735)

	Any PAC		Basic PAC		Comp PAC	
	aOR^a^	95% CI	aOR^a^	95% CI	aOR^a^	95% CI
Age (years)						
15–19	1.00	1.00, 1.00	1.00	1.00, 1.00	1.00	1.00, 1.00
20–24	0.93	0.60, 1.44	0.87	0.58, 1.31	0.63***	0.47, 0.85
25–29	1.06	0.71, 1.57	0.98	0.69, 1.41	0.85	0.59, 1.21
30–34	1.00	0.61, 1.66	1.00	0.69, 1.45	1.07	0.66, 1.73
35–39	0.78	0.49, 1.25	0.85	0.62, 1.18	1.01	0.73, 1.40
40–44	0.86	0.53, 1.42	0.98	0.68, 1.43	1.05	0.74, 1.50
45–49	0.82	0.46, 1.46	0.85	0.52, 1.38	0.94	0.59, 1.49
Education						
Never	1.00	1.00, 1.00	1.00	1.00, 1.00	1.00	1.00, 1.00
Primary	1.26	0.80, 2.01	1.09	0.71, 1.68	1.17	0.72, 1.88
Secondary	1.55	0.79, 3.04	1.39	0.84, 2.28	1.36	0.88, 2.12
Higher	1.00	1.00, 1.00	1.91	0.63, 5.79	1.81*	0.91, 3.60
Residence						
Rural	1.00	1.00, 1.00	1.00	1.00, 1.00	1.00	1.00, 1.00
Urban	3.18	0.28, 35.90	2.84	0.69, 11.68	2.18	0.58, 8.23
Wealth tertile						
Poorest	1.00	1.00, 1.00	1.00	1.00, 1.00	1.00	1.00, 1.00
Middle	4.09***	1.54, 10.88	2.38*	0.99, 5.72	2.44	0.58, 10.21
Wealthiest	46.42***	4.18, 515.75	13.16***	3.07, 56.43	6.41**	1.43, 28.77

*P < 0.10, ^**^P < 0.05, ^***^P < 0.01.

a Adjusted odds ratio.

## Discussion

Findings indicate insufficient availability, readiness and accessibility of PAC services in both Nigeria and Côte d’Ivoire. These conclusions are consistent with research conducted in other sub-Saharan African countries, showing critical gaps in PAC services, including availability and signal functions across 10 countries (Owolabi *et al.*, 2019). Only 48.4% of facilities with the capacity to provide basic PAC in Nigeria are providing it, which is mostly driven by a lack of services in primary public facilities. In Côte d’Ivoire, PAC service availability is much higher at 70.5%, as public primary care facilities are more likely to offer these services. Fewer facilities have the capacity to provide comprehensive PAC services, but when they have the capacity, a higher proportion of them do.

On examining PAC signal functions, as a dimension of quality of care, we found that approximately one-third of facilities in Nigeria that had the capacity to provide basic PAC had all signal functions, and 36.9% met these criteria in Côte d’Ivoire. However, a more detailed measure of the percentages of signal functions available provides a more nuanced picture, as facilities providing PAC had ∼90% of necessary components, and those that did not provide PAC at the time of the survey already had approximately half of all signal functions. Lack of training of primary care providers was a critical barrier in public health settings in Nigeria, while lack of medication (oxytocics) and intravenous fluids was a concern in both settings. Training and these commodities are also essential in providing EmOC following delivery, indicating a need for efforts to strengthen the capacity of primary care facilities to provide EmOC, including PAC, to reduce mortality and prepare for the transfer of more complicated cases.

These findings emphasize the need for improvements in primary care services more broadly, which aligns with existing research highlighting deficiencies in the availability, quality and coverage of maternal care and related services. Investigators determined that 46% and 27% of facilities in Kenya had high signal function scores related to routine delivery care and EmOC, respectively; these numbers were 18% and 5% in Namibia (Diamond-Smith *et al.*, 2016). A 2013 study from Burkina Faso, Ghana and Tanzania among only rural, primary health facilities found somewhat higher levels of readiness for antenatal and childbirth care, with facilities having 65% of basic EmOC signal functions. However, none of the primary facilities in Burkina Faso and Ghana had the capacity to remove retained products of conception (Duysburgh *et al.*, 2013). Researchers have also observed low levels of effective coverage, i.e. quality-adjusted service coverage metrics, of a number of primary care services in high mortality countries, including antenatal, family planning and sick-child care (Leslie *et al.*, 2017). This suggests the inadequacies we observed in relation to basic and comprehensive PAC in Nigeria and Côte d’Ivoire are likely indicative of more extensive deficiencies in primary care service availability, quality and use. This has implications for maternal health outcomes as high maternal mortality ratio settings are unlikely to be able to achieve reductions in maternal deaths amid continued lack of quality EmOC, among which PAC is a critical, life-saving element.

While approximately 80% of women in Nigeria and Côte d’Ivoire lived within 10 km of a facility that provides PAC, results show significant inequities in accessibility related to education, residence and wealth. These social inequities result in the most vulnerable women—who are the most likely to experience an unsafe abortion and thus have the greatest need for PAC (Bell *et al.*, 2020a,c)—having the poorest access to basic and comprehensive PAC. This has the potential to manifest in disparities in outcomes, which is consistent with existing evidence that poor, rural women are most likely to experience abortion-related severe morbidity and mortality (Singh *et al.*, 2018). Although we did not directly measure the potential need for and use of PAC services among all women in our investigation, the aforementioned prior research suggests that the need for PAC is greatest among those who we found to be least likely to live near a facility offering these services. We thus view observed differences in PAC access as more than inequality; they reflect horizontal inequity in healthcare access related to women’s sociodemographic characteristics (O’donnell *et al.*, 2007).

This study has several strengths. To our knowledge, this is the first large-scale assessment of public and private sector PAC service availability and accessibility in these countries, addressing the dearth of information on this essential EmOC service. With regard to measurement, we provide data on the availability of specific components necessary for basic and comprehensive PAC service provision, in addition to creating a more nuanced measure of readiness via a composite signal functions index. This allowed us to better assess specific signal functions that are lacking and the level of remaining work that is required for all facilities to meet these readiness criteria. Additionally, we were able to link the facility data to population-based data, allowing us to assess structural disparities in access to PAC. This unique feature of our data further addresses the question of why we see such marked inequities in the distribution of maternal mortality and morbidity among rural, poor women.

However, this study is not without limitations. We had relatively small facility sample sizes in both countries, particularly Côte d’Ivoire, which makes our results more sensitive to biases as a result of sampling variation. Additionally, the SDPs were selected based on the facilities that serve a nationally representative sample of reproductive-age women; they were not sampled from a facility sampling frame to be nationally representative of facilities. We constructed weights that account for the probability of selection and response rate within each facility type and state for public facilities in Nigeria and facility type in Côte d’Ivoire using the latest available sampling frame, but the accuracy of the probability of selection relies on two assumptions: (1) that the sampling frame is correct and (2) that the facilities included in the sample are representative of the other facilities of a given type within a specific sampling geography. For the private facilities in Nigeria, facilities that serve more populated areas were more likely to be selected given the probability proportional to size sampling design. Thus if these facilities are systematically different from those serving less populated areas, the second assumption may not hold.

While our readiness score is an attempt to offer a more nuanced assessment of the potential for facilities to provide quality PAC services than the ‘all or nothing’ measurement approach, it fails to indicate what proportion of women needing PAC can be safely treated in the context of existing signal functions and what proportion of facilities are equipped to treat severe maternal morbidity. Some signal functions are curative, while others are preventive. A more in-depth analysis of PAC service readiness in relation to severe maternal morbidity, maternal near miss and maternal mortality would be important to understand how the readiness score predicts abortion-related outcomes.

With regard to evaluating access, we were limited in the specificity with which we could link women to facilities based on ethical review board restrictions on the use of GPS data in Nigeria. By assigning women’s location as the centre point of their EA, we lost nuance in determining women’s distance to facilities within EAs. However, in Côte d’Ivoire, where we ‘did’ have access to individual-level GPS, the results were very similar when using the individual versus EA centre GPS points. Given the conclusions were the same, we chose to use the same analytic approach in Côte d’Ivoire and Nigeria for consistency.

There were also limitations in our evaluation of facility PAC readiness. While PAC signal functions focus on the availability of individual service/commodity availability and not on specific provider training, our data suggest lower availability of a provider formally trained in PAC than there were facilities providing PAC. This indicates that some facilities may have been relying on providers who may not have been formally trained in PAC. However, this discrepancy may stem from confusion around the meaning of ‘formal’ training as providers may receive on-the-job training from more experienced providers but may not view this as ‘formal’ training; this was a limitation of the way we asked the question. In general, the way we measured readiness would tend to overestimate the true readiness since we asked about general availability of some of these services, devices, and medicines, which actually may not have been available on the day of the survey. Finally, while this study provides important information on service availability and readiness, we did not aim to capture the quality of care more broadly, including cost, technical competency, interpersonal interactions and outcomes.

Despite these limitations, our study findings have important implications. Results provide stakeholders with country-specific actionable information in their efforts to improve PAC service readiness in order to prevent unnecessary maternal morbidity and mortality. Specifically, we see the opportunity to greatly improve the number of facilities—particularly public primary facilities—offering basic PAC services. This could help to reduce the significant structural disparities in access to life-saving PAC services that we observed in both countries. Our results show that many facilities that do not already offer PAC have several of the basic signal functions and could meet readiness criteria with limited additional tools or training. Among facilities already offering PAC, while many do not have all basic PAC signal functions, most are missing very few signal functions. However, more substantial improvements would be required to meet comprehensive PAC readiness criteria. Advocacy efforts and in-country champions should seek to increase political will towards improving the availability of this essential healthcare service and human resources in the public sector more broadly, particularly at the primary facility level. In Nigeria, these efforts should target federal, state and local governments, which hold a lot of power in managing the public health system, while stakeholder engagement in Côte d’Ivoire should focus nationally as the public health system is more centralized. Improving this critical component of EmOC at primary facilities—and primary healthcare generally—could greatly benefit women in both countries, particularly the most disadvantaged who are currently least able to access these essential services.

## Conclusion

Our study found that PAC service availability and readiness are poor in two West African countries. Additionally, the results highlight inequities in access to PAC services, including facilities with readiness to provide basic or comprehensive PAC, illustrating one of the sources of inequities in postabortion morbidity and mortality. Addressing facilities’ service readiness will improve the quality of PAC provided and ensure postabortion complications can be treated in a timely and effective manner, while expanding the availability of services to additional primary-level facilities would increase access—both could help to reduce avoidable abortion-related maternal morbidity in these settings that is primarily experienced by disadvantaged women. More broadly, our findings signal that facilities in these settings are in need of a number of improvements to maternal primary care. Implementing training programmes to strengthen providers’ capacity in maternal services and improving facilities’ stock of essential medicines and devices will help strengthen the primary care response and with it PAC capacity.

## Data Availability

The data underlying this article include PMA Cote d’Ivoire Round 2 female (doi: 10.34976/716t-1697) and service delivery point data (doi: 10.34976/nd0a-ew46) and Nigeria Round 5 female (doi: 10.34976/b5eg-a867) and service delivery point data (doi: 10.34976/xjzj-y288), which are available at www.pmadata.org/data/request-access-datasets. The modified GPS dataset for Cote d’Ivoire (doi: 10.34976/pzx0-8x92) is also available, however, the in-country ethical review board for Nigeria does not permit GPS data sharing.
