# Erratum to: postabortion care availability, facility readiness, and accessibility in Nigeria and Cote d’Ivoire

**DOI:** 10.1093/heapol/czab082

**Published:** 2021-07-10

**Authors:** Suzanne O Bell, Mridula Shankar, Saifuddin Ahmed, Funmilola OlaOlorun, Elizabeth Omoluabi, Georges Guiella, Caroline Moreau

In the originally published version of this manuscript, the Funding section included additional text from correspondence in error.

This error has been corrected online.

