# Correction to: Postabortion care availability, facility readiness and accessibility in Nigeria and Côte d’Ivoire

**DOI:** 10.1093/heapol/czac066

**Published:** 2022-08-12

This is a correction to: Suzanne O Bell, Mridula Shankar, Saifuddin Ahmed, Funmilola OlaOlorun, Elizabeth Omoluabi, Georges Guiella, Caroline Moreau, Postabortion care availability, facility readiness and accessibility in Nigeria and Côte d’Ivoire, Health Policy and Planning, Volume 36, Issue 7, August 2021, Pages 1077–1089, https://doi.org/10.1093/heapol/czab068

In the originally published version of this manuscript, the fourth sentence of the first paragraph read: ‘In high-resource settings, the abortion rate decreased significantly by 31% from 1990-94 to 2015-19, whereas the corresponding rates in low-resource settings were similar in both periods (Bearak et al., 2020) to 27 in the 2010-14 period, whereas the corresponding estimates in low-resource settings were 39 and 37, respectively.’

The correct version of the sentence is: ‘In high-resource settings, the abortion rate decreased significantly by 31% from 1990-94 to 2015-19, whereas the corresponding rates in low-resource settings were similar in both periods (Bearak et al., 2020).’

These details have been corrected only in this correction notice to preserve the published version of record.

